# Total care expenditures and their drivers among older adults: A study on health and long-term care expenditures in South Korea

**DOI:** 10.1186/s12913-022-07977-5

**Published:** 2022-04-25

**Authors:** Hyo Young Lee, Young-Ran Chin

**Affiliations:** 1 Department of Health Administration, Dongseo University, Busan, South Korea; 2 Department of Nursing, Chungwoon University, Chungnam, South Korea

**Keywords:** Care needs level, Long-term care (LTC), Health care, Associated factors, Characteristics, Older adults

## Abstract

**Background:**

South Korea operates two different national insurance systems: health care insurance covers medical services and long-term care (LTC) insurance covers residential care and home care services. Total care expenditures include benefits from both these insurance schemes and personal payments made for receiving these services. This study aims to identify total care expenditures per older person along with related factors and their effects on care expenditures.

**Methods:**

We analyzed claims data of 2017 for LTC and health care insurance in Korea using multiple regression analysis. Participants were recipients of LTC insurance, aged 60 years or above (n = 650,059). The variables of interest included socioeconomic characteristics, disabilities, chronic diseases, and care needs levels.

**Results:**

The total expenditures were approximately USD 9,808,922,016 for 650,059 older people (USD 15,089.28 ± 8,006.57 per person) in 2017. The benefits of national health insurance accounted for 86.03% of the total, while personal payments accounted for 13.97%. Comparing the expenditure across services, the total amount was found to be much higher for LTC services. The personal payments were similar for the two insurance schemes, and the proportion of expenses by service type (to total expenses) was greater for LTC (LTC versus health care expenditures: 63.25% versus 36.15% of the total expenditures). The total care expenditures differed significantly according to recipient characteristics. Older adults who were women, between 75–84 years old, with higher care needs levels, and who suffered from diseases and lived in the residential facilities were associated with an increase in total expenditures. Moreover, factors such as any type of disability and living alone were related to a decrease in total care expenditures.

**Conclusions:**

The increase in care expenditures should be monitored from an integrated perspective on overall health care and LTC, and to reduce care needs. In addition, we should focus on the factors involved in using (receiving) services for older individuals and complementing the lack of or inadequate services to enhance and sustain the LTC and health care service systems. Older adults receiving full basic livelihood security and living alone should receive greater attention from the perspective of social equity.

## Background

South Korea is facing a huge demand for medical and long-term care (LTC) services, given the increasing population of older adults. The country reported the highest number of doctor consultations per person in 2017 among Organisation for Economic Co-operation and Development (OECD) countries, with 16.6 times a year, compared to the average of other OECD countries [1]. To proactively meet the growing demand for care, South Korea introduced two social insurance schemes: National Health Insurance Service (NHIS) in 1977 (https://www.nhis.or.kr) for medical services; and Long-term Care Insurance (LTCI) in 2008 (https://www.longtermcare.or.kr) for LTC services [2]. NHIS and LTCI are Korean social security systems that cover all citizens except for public assistance recipients, and operate in the form of insurance. NHIS provides benefits for disease, diagnosis, treatment, and rehabilitation, whereas LTCI provides home care (visit care, visit nursing, short-term respite care, visit bathing, day and night care), residential facility care, welfare equipment, and cash benefits [2].

When discussing a country’s health expenditure, it is essential to consider both the medical and care costs. In particular, in the case of older adults, care and medical costs will increase with time, hence, it is necessary to examine these two costs jointly. LTC was introduced to reduce medical costs and to use resources more efficiently. Given that considerable time has passed since its introduction, an investigation of the current status of the overall health expenditure is required, along with exploring related factors to ensure it is cost-effective and sustainable.

Regarding the assessment of care needs levels for LTC in South Korea, registered nurses (RNs), physical therapists, or occupational therapists in the NHIS provide ratings based on various criteria, which are followed by the computation of the long-term care approval score for the judgement rating. The checklist for long-term care needs levels includes applicants’ medical and functional state with regard to personal needs and environment, general status, physical function (how much help they needed from other people during the previous month), social function, cognitive function, behavior problems, medical treatment (symptoms during the previous two weeks), and rehabilitation needs (disability and joint problems) [3]. Medical and LTC services in South Korea have been provided universally as social insurance, and there is no price competition because the care benefits and prices are set by NHIS [4].

LTCI provides standardized care according to the recipients’ care needs levels [3]. The amount of medical services may increase depending on the amount of LTC services, and conversely, if someone has received proper LTC services, the amount of medical services may be reduced. Feng et al. reported that LTC services reduce medical care costs [5], and more care costs are needed at a higher level [6, 7]. Medical care expenditure was also associated with many factors, such as the types and number of diseases, subjective health conditions, and characteristics of people. At the macro level, the healthcare system, the payment system, and the economic status, among others, influence expenditure levels [8].

LTC expenditures may arise from the complex interaction of several determinants, which is primarily the care needs level of each recipient. One of the main factors influencing care expenditures is the care needs level, which reflects the range of medical needs, physical functions, and social care needs [6, 7]. The recipients of LTC services receive certain amounts of services depending on their care needs level [3]. Knapp et al. indicated that dependency of care recipients is very sensitive to LTC expenditures [9]. LTC expenditures were also found to be influenced by the type of service and whether the services were delivered universally [4]. Costa-Font et al. reported that age and demographic composition are the most essential factors influencing the expenditures of LTC services [10]. Existing studies related to care expenditures among older adults have been based on some related factors such as physical activities, demographic shift, or prevalence of chronic disease, but they have merely focused on medical expenditures [11, 12] or LTC expenditures alone [6, 7]. Lum et al. studied LTC expenditures with a focus on specific diseases such as mental disorders [13]. LTC services evidently reduce hospital stay and medical expenditures, but they could be substitutes for medical services [5]. Therefore, if we want to determine total care expenditures, it is necessary to examine both medical and LTC expenditures as well as to identify the factors influencing them. This study investigated the level of total care expenditures—LTC and health care expenses—per year for one older adult and identified the related factors, such as socioeconomic characteristics, LTC level, and disease-related aspects, along with their impacts on care expenditures.

## Methods

### Study population and variables

The older adult beneficiaries of the two insurance schemes from January 1 to December 31, 2017 (n = 650,059) accounted for 8.9% of the total older adult population. In this study, “total care expenditures” denote benefits from the two insurance schemes and personal (out-of-pocket) payments made for receiving these services. The variables of interest, which were related to total care expenditures, included socioeconomic characteristics, level of care needs, disabilities, and chronic diseases. The socioeconomic characteristics included sex (male or female), age group (60–64, 65–74, 75–84, 85–94, or above 95), economic status (dependent on full basic livelihood security, dependent on partial basic livelihood security, or general) and cohabitants (spouse, family member, relative or neighbors, residential facility or caregivers, volunteers, etc.).

The LTC levels for people ranged from Levels 1 to 5, with Level 1 denoting the highest care needs, and Level 5 denoting the lowest. The LTC levels were measured by the care needs of the LTC service users. The level assessment procedures included receiving long-term care needs scoring and deciding on the level. The five areas of assessment used to score LTC included: daily life function (12 items), cognitive function (10 items), behavioral change (22 items), nursing care (10 items), and rehabilitation (10 items). From the LTC recognition score is derived a 100-point translation score, the first grade is 95 points or higher (a person who is completely dependent on the help of another person to go about their daily life), the second grade is between 94 to 75 points (a person who is mostly dependent on the help of another person to go about their daily life), the third grade is from 74 to 60 points (a person who is in partial need of the help of another person to go about their daily life), the fourth grade is between 59 to 51 points (a person with mental and physical disabilities and who is in partial need), and the fifth grade is between 50 to 45 or lower (a person with dementia).

Disabilities diagnosed by a medical doctor were divided into five categories—physical, cerebral, visual, hearing, and complex or other—considering the frequencies of the occurrence of the disabilities. This disability classification was based on South Korean ‘enforcement decree of the act on welfare of persons with disabilities', and complex or other disabilities were classified as one category because a small proportion of those receiving long-term care [14]. We considered 14 types of diseases under chronic diseases (including a category for no disease), in accordance with the major diseases reported in medical diagnosis among LTC beneficiaries. As for chronic diseases of older adults, the most LTC-affected diseases were identified by a doctor’s diagnosis.

### Data analysis

The NHIS and LTCI claims data for LTC and health care insurance for the year 2017 were merged by an LTCI client identifier for the calculation of total care expenditures. The data used in this study were analyzed by researchers using customized LTC care database built by the NHIS. The data were anonymized so that personal information could not be identified by default, and were provided for analysis to approved researchers only in an NHIS data analysis room. Total care expenditures per person were calculated, including the total care expenditures of LTC recipients, by adding up all the costs of receiving LTC and medical services. The expenditures were also calculated for each characteristic of the older adults, and the statistical significance of expenditures was tested through analysis of variance (ANOVA) with least significant difference (LSD). Multiple regression analysis was used, controlling for covariates, to investigate the factors influencing the expenditure of care. They were mutually controlled by the variables in the analysis model (sex, age, economic status, LTC care needs level, types of disabilities, cohabitants, and types of diseases). This study was approved as per the ethics guidelines of the institutional review board (IRB; D University approval number: 10141493-E-2019–01).

## Results

**Fig. 1:**
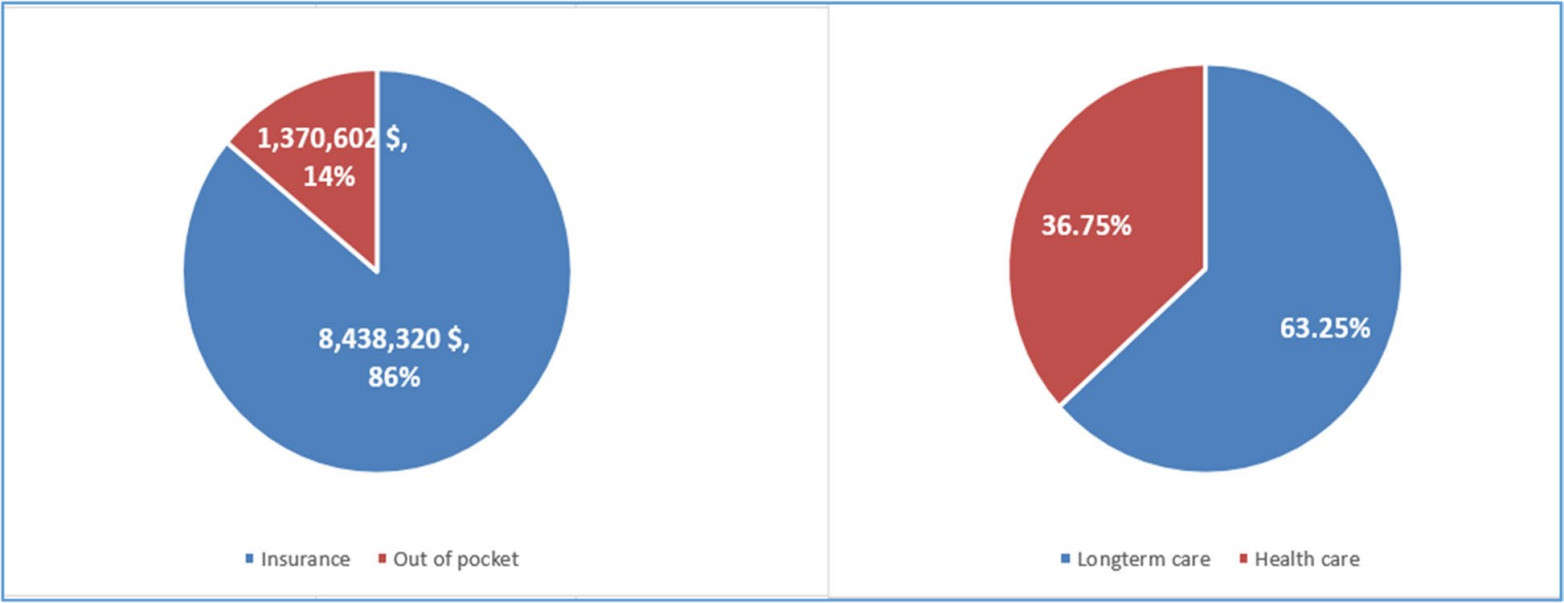
The total expenditures and proportion of health care and long-term care (LTC) for older adults

Table 1 presents the total care expenditures (including medical and LTC expenditures) of all LTCI recipients in South Korea in 2017. Benefits from the two insurance schemes accounted for approximately 86% of the total care expenditure, while personal (out-of-pocket) payments accounted for approximately 14%. The total expenditure on LTC services was approximately 1.72 times more than that on health care services, and the proportion of LTC insurance benefits (to total LTC expenditures) was approximately 7.5% higher than that of health care insurance benefits (to total health care expenditures; Fig. 1, Table 1). Regarding personal payments, the proportions were considerably higher in medical services, but the amounts of personal payments were similar in the two services (Table 1).

**Table 1:** Total expenditures on health care and LTC services for 650,059 older Korean adults in 2017

		**Total**	**Insurance** **Benefits**	**Personal payment** **(co-payments)**
**Net total expenditure in 2017** **(USD)**	**Total**	9,808,922,016 (100.00%)	8,438,320,357 (86.03%)	1,370,601,659 (13.97%)
	**Long-term care**	6,203,876,483 (63.25%)	5,512,429,529 (88.85%)	691,446,955 (11.15%)
	**Health care**	3,605,045,533 (36.75%)	2,925,890,828 (81.16%)	679,154,705 (18.84%)
Per capita expenditure* in 2017 **(USD)**	**Total**	15,089.28 ± 8,066.57	12,980.85 ± 8225.82	2108.43 ± 1953.83
	**Long-term care**	9543.56 ± 5100.05	8479.89 ± 5900.53	1063.67 ± 986.29
	**Health care**	5545.72 ± 8144.03	4500.96 ± 8367.15	1044.76 ± 1832.06

^*^ Per capita expenditure (total expenditure per person) is the value arrived at by dividing the total expenditure by the number of people

Total care expenditure differed significantly according to socioeconomic characteristics (sex, economic status, age groups, and cohabitants), care needs level, and type of disability and disease as determined by ANOVA with LSD (Table 2). Persons who were male, 74 years of age or less, and “dependent on full basic livelihood security” reported higher total care expenditures than others. With regard to the level of care needs for LTC, level 1 had the highest care expenditure and it was statistically significant. Concerning disability type, persons with “complex disability and others” had the highest expenditure of care; regarding cohabitants (or type of residence), people living in a facility or living with people other than family members or relatives (“Other:” caregivers, volunteers, etc.) showed the highest expenditure of care. Among diseases, diabetes and cancer showed the highest expenditure of care (Table 2).

**Table 2:** Total health and LTC expenditures by characteristics of older adults in 2017

**Characteristics**		**% of N**	**Total care expenditures**			
			**Mean** **(USD)**	**SD** **(USD)**	**Between group comparison**	**LSD**
	Total	650,059	15,089.28	8,066.57		
Sex*	(a) Male	26.8	**15,462.13**	9,433.11	t = 509.533 *(p* < *0.01)*	a > b
	(b) Female	**73.2**	14,952.59	7,498.89		
Age*	(a) 60–64	5.0	**16,970.68**	10,486.67	F 4,650,054 = 2447.275 *(p* < *0.01)*	a > b > c > d > e
	(b) 65–74	16.9	16,703.98	10,262.26		
	(c) 75–84	**46.6**	15,004.61	7,736.72		
	(d) 85–94	29.1	14,063.26	6,447.48		
	(e) Above 95	2.4	13,897.00	5,878.48		
Economic status*	(a) Dependent on full basic livelihood security	18.1	**15,781.39**	7,288.44	F 2,650,056 = 583.940 *(p* < *0.01)*	a > b > c
	(b) Dependent on partial basic livelihood security	19.8	14,734.95	7,857.08		
	(c) General	**62.1**	15,000.00	8,332.94		
Care needs level*	(a) 1	4.2	**21,209.58**	10,929.88	F 4,650,054 = 10,074.386 *(p* < *0.01)*	a > c > d
	(b) 2	10.3	18,246.03	8,450.44		a > b > e
	(c) 3	34.7	15,369.20	7,704.04		
	(d) 4	42.9	13,272.05	6,909.57		
	(e) 5	8.0	10,879.08	5,693.55		
Disability*	(a) None	59.1	14,337.73	6,946.24	F 5,650,053 = 8244.702 *(p* < *0.01)*	f > b > d
	(b) Physically handicapped	15.1	14,839.00	7,572.52		f > c > a > e
	(c) Cerebral	15.0	16,774.51	9,323.43		
	(d) Visual	3.1	14,376.93	7,699.10		
	(e) Hearing	4.8	13,994.97	6,970.44		
	(f) Complex disability and others	3.0	**25,096.14**	15,064.99		
Cohabitant*	(a) Alone	19.7	12,906.55	6,457.00	F 5,650,053 = 20,119.885 *(p* < *0.01)*	f > e > b > d > a
	((b) Spouse (husband or wife)	23.0	14,290.66	8,861.02		b > c
	(c) Family member	32.2	13,213.33	7,625.53		
	(d) Relative or/and neighbors	0.8	13,886.07	7,541.12		
	(e) In a residential facility	18.4	**19,541.51**	4,549.63		
	(f) Other	5.9	**22,050.31**	10,978.99		
Disease*	(a) Dementia	27.7	14,867.18	6,604.44	F 8,650,051 = 1488.488 *(p* < *0.01)*	c > a
	(b) Cerebrovascular disease (CVD)	16.9	16,307.94	9,274.41		b > d > k
	(c) Dementia + CVD	5.4	16,966.68	8,282.91		i > e > c > a > d > g
	(d) Hypertension	1.4	13,930.41	8,151.74		i > e > j > k
	(e) Diabetes mellitus	1.7	**18,600.01**	12,059.80		i > e > j > g
	(f) Arthritis	9.7	13,144.28	6,445.92		
	(g) Back and hipbone pain	13.7	13,424.40	6,723.18		
	(h) Fracture and dislocation	8.9	14,880.83	7,488.59		
	(i) Cancer	1.7	**18,975.52**	13,422.60		
	(j) Dyspnea	0.6	15,101.01	9,682.01		
	(k) Hearing loss	0.2	11,303.07	6,681.18		
	(l) Cataract	1.2	13,051.61	6,975.54		
	(m) Other	9.2	16,186.11	9,866.33		
	(n) None	1.8	15,720.94	7,830.13		

SD: Standard deviation, LSD: least significant difference, *statistically significant at *p* < 0.01, bold numbers were the highest expenditure within each characteristic

Table 3 shows the results of the regression analysis after controlling for covariates (mutually controlled by the variables). Individuals who were female, 75–84 years of age, of “general” economic status, and with higher care needs were significantly related to the increase in total care expenses. In addition, the number of people with disabilities was associated with a decrease in total care expenditures compared to people without disabilities. Regarding cohabitants (or type of residence), the highest expenditure of care was in the case of living in a residential facility and with others (e.g., caregivers and volunteers). Furthermore, dementia, cerebrovascular disease (CVD), back and hipbone pain, and fracture and dislocation were particularly associated with high care expenditures. In Table 2, total care costs differed according to types of disabilities, and “complex disabilities and others” seemed to incur the most expenditures. However, in Table 3, total care expenditures decreased for all the disabilities, with the lowest total care expenditures reported for “complex disabilities and others” calculated using multiple regression analysis controlling for all the variables used.

**Table 3:** Factors associated with total care expenditures: After controlling for co-variates

**Variables**		**β**	**Standard error**	***P*-value**	**95% CI**	
					**Lower**	**Upper**
	**Constant**		0.056	.013	.029	.250
**Sex** (ref: male)	**Female**	**.005**	0.013	.000	.065	.117
**Age**	**65–74**	-.001	0.028	.449	-.076	.034
(ref.: 60–64)	75–84*	**.012**	0.027	.000	.135	.242
	85–94*	**-.026**	0.029	.000	-.504	-.391
	Above 95*	**-.023**	0.044	.000	-1.235	-1.061
**Economic status**	Partial basic livelihood*	**.604**	0.018	.000	11.653	11.723
(ref.: full basic)	General*	**1.053**	0.015	.000	16.710	16.769
**Care needs level**		**.020**	0.006	.000	.147	.171
**Disability**	Physically handicapped*	**-.016**	0.016	.000	-.377	-.314
(ref.: none)	Cerebral*	**-.032**	0.020	.000	-.740	-.663
	Visual*	**-.007**	0.035	.000	-.383	-.247
	Hearing*	**-.002**	0.026	.001	-.138	-.037
	Complex or others*	**-.056**	0.033	.000	-2.569	-2.440
**Cohabitant**	Spouse (husband or wife)*	**.016**	0.019	.000	.256	.331
(ref.: alone)	Family member*	**.008**	0.017	.000	.095	.162
	**Relative or/and neighbors**	.000	0.062	.991	-.121	.122
	In a residential facility*	**.046**	0.020	.000	.869	.947
	Other*	**.072**	0.027	.000	2.298	2.403
**Disease**	Dementia*	**.037**	0.042	.000	.551	.715
(ref.: none)	CVD*	**.037**	0.043	.000	.680	.849
	Dementia + CVD*	**.029**	0.047	.000	.882	1.066
	Hypertension*	**.009**	0.062	.000	.454	.695
	DM*	**.013**	0.058	.000	.644	.873
	Arthritis*	**.016**	0.044	.000	.338	.511
	Back and hipbone pain*	**.035**	0.043	.000	.700	.869
	Fracture and dislocation*	**.033**	0.044	.000	.814	.988
	Cancer*	**-.044**	0.058	.000	-2.766	-2.538
	Dyspnea*	**.014**	0.081	.000	1.251	1.570
	**Hearing loss**	.001	0.014	.074	-.024	.531
	Cataract*	**.002**	0.068	.047	.002	.268
	**Other**	.002	0.044	.255	-.036	.137

R^2^ = 0.683, F 32,650,026 = 43,707.73, *statistically significant at *p* < 0.001,bold numbers were statistically significant

## Discussion

This study identified the level of total care expenditures per year per older person and explored whether the total expenditures vary by socio-economic factors, care needs levels, and type of disability and disease, along with the effects of these factors on total care expenditures. The results reveal that, in 2017, the total care expenditures were USD 15,089.28 (person/year), the total expenditure on LTC services was approximately 1.72 times more than that on health care services, and the amounts of personal (out-of-pocket) payments were similar for the two service types. Older persons who were female, between 74–84 years old, of general economic status, without disabilities, not living alone, and with certain chronic diseases were associated with an increase in total care expenditures. The total expenditure in the case of those living with family members, including spouses, was higher than that of adults living alone. Future studies should examine if there is an unmet care need for adults living alone, in terms of health equity. Furthermore, living in LTC facilities was related to an increase in their total expenditures. Thus, we should ensure that community care can meet their needs, and if possible, try to reduce expenditure by delivering home or community care services. It will also function as a strategy to improve the quality of life of older adults. Some studies have reported that home and community care services have reduced the care needs of older adults [15, 16]. Besides, Kuzuya et al. reported that community care was associated with a lower mortality rate among frail older people and Chang et al. indicated that community care improved patient health outcomes [17, 18].

The total expenditures per person (for one year) calculated in this study were USD 15,089.28 ± 8006.57, which represented 70.04% of the Korean annual minimum salary of USD 21,543.72 [19]. Considering that the total annual medical expenses per capita per year in Korea were about USD 1,306 in 2018, the care expenses of LTC recipients were nearly 11.6 times higher than that of the total Korean population. The total annual medical expenses per capita of older adults per year were approximately USD 3,884, and the care expenses of LTC recipients were also approximately 3.9 times higher than that of the total population of Korean older adults [2]. According to OECD statistics, OECD countries used an average of 1.5% of their Gross Domestic Product (GDP) for total LTC services in 2017, and an average of 8.8% of their GDP for health services, and the expenditure is continuously increasing [20, 21]. Therefore, it is believed that various strategies are needed to reduce the increase in care expenditures due to the growing older population. Especially, to reduce the cost of care for people at level 1, efforts will need to be made to prevent the increase of care needs by providing home care or community care at an early stage through early detection. It will also require multi-level efforts to maintain and promote the health of older adults through various community programs.

Among the socio-economic factors, being female was associated with an increase in total expenditures after controlling for covariates, which would be due to women’s lower chances of recovery compared to men [22]. The 75–84 age group revealed the highest total care expenditures compared to other age groups, but the age of over 85 years revealed less total care expenditures compared to those below 64 years. Therefore, we need to investigate more the areas of care that lead to an increase in expenditures and find ways to reduce expenses. In this context, comprehensive care will have to be used to reduce total care expenditures throughout the life of an individual. From an early period, older adults’ care needs should be assessed and proper LTC services should be provided, if applicable; moreover, we should also ensure that LTC services are accessible to those who need them. Home care-based comprehensive services will be needed to delay deterioration, especially in the case of those with care need levels 4 or 5, using a variety of local resources to discover the needs of the individuals [23]. Currently, pilot projects for comprehensive community care are underway in South Korea. Thus far, housing, health care, welfare, and care services have been supplied separately by the respective government departments. However, comprehensive community care is planned for providing integrated services through efficient use of resources. Therefore, it could reduce care needs and care expenditures and improve the quality of life of the older adult population [24].

Regarding economic status, people with partial basic livelihood security and of general status were associated with an increase in total expenditures compared to those with full basic livelihood security. The results are confirmed by the fact that people with more economic power can pay more for overall medical and LTC services. Future research should carefully identify and examine the kinds of services needed to meet the needs of those who receive basic livelihood security. On the one hand, LTC recipients with disabilities were associated with a decrease in total expenditures compared to normal LTC recipients, which indicates the need to examine whether there is a problem with accessibility in care use due to which the total expenditure of care is reduced. On the other hand, it was found that recipients with a disability did not necessarily experience an increase in total care expenditures. These two possibilities will have to be confirmed in future studies, along with the accessibility of both LTC and health care services.

According to Jin et al., adults who are female, facility service users, with higher care need levels, and with lower income faced high LTC expenditures [7]. Moreover, in regions with more households with older adults who are living alone and are 75 years old, more nursing homes and doctors per 1,000 older adults, the LTC expenditures were found to be high. As for medical expenditures, disease prevalence, service utilization, service prices, and demographic reasons were influential factors leading to rising expenditures [12]. Lin et al. also reported that people who had higher care needs levels were women and of older age, and were related to higher LTC expenditures [6].

The finding that the care needs level increases the total care expenditures are similar to the results of previous studies related to LTC expenditures [6, 7], but the relevance of the care needs level and total care expenditures was much lower than that of other factors in increasing the total care expenditures. This shows that the overall expenditure exhibits greater influence due to economic status, cohabitants, and diseases. In addition, several diseases in this regard were associated with an increase in total expenditures, but in older adults with cancer, the total care expenditures tended to decrease. It is believed that the total care expenditures increase due to other factors for people suffering from cancer, rather than the burden of the disease itself. In the case of patients with cancer, in the medical service, it is believed that it is the payment system that lowers the insurance price and requires merely 5% personal payment, and the increased guarantee for this part might have led to the lowering of the total care expenditures [2]. Currently, there is no benefit limit to health care, but each LTC level has a benefit limit (monthly maximum coverage); the higher the care needs level, the higher the limit [3]. It can be said that in the case of a higher level, the total care expenditures tend to be high. South Korean LTCI takes into account financial stability and appropriate care levels, and it currently provides USD 1,280 per month for Level 1, USD 1,138 for Level 2, USD 1,091 for Level 3, USD 1,002 for Level 4, and USD 861 for Level 5. It is important to consider the financial stability of LTCI and care needs, while at the same time provide people with the necessary care services. Besides, we should continue to make efforts to sustain the care system through future studies.

As care needs increase, LTC and medical service supply increases, and consequently, the total expenditure increases. We are facing financial challenges that require a care system that develops along with the growing older population, and at the same time, leads to lower financial requirements. We need to investigate the areas of care that lead to an increase in expenditures and find ways to reduce expenses. In this context, comprehensive care will have to be used to reduce total care expenditures and delay older adults’ care needs during the early period of older adulthood. Currently, South Korea provides services according to the LTC level to all its citizens without a means test in the LTCI [25]. However, differentiating benefits or co-payments through means tests may also be a way to secure financial stability. In addition, in South Korea, laws mandate that service institutions be evaluated periodically and incentives offered to LTC service institutions to improve the quality of their services [25]. As such, along with the increase in the amount of LTC services offered, quality evaluation should be continuously monitored so that quality service can be provided.

This study has several limitations. First, depending on the time or duration of evaluating LTC needs depending on the participant, the expiration date of the LTC needs level that was not come or the person who did not apply for re-rating of their LTC needs level may have been excluded from the study. In future studies, everyone could be included for evaluating LTC needs at least twice. Second, the socioeconomic characteristics of the participants in 2017 were investigated, and could only be analyzed within a limited time and space due to personal information protection restrictions; moreover, various related factors were not examined due to the limitations of variables. Third, the comorbidity of older persons may be a result that was not reflected in this study. Finally, the data used for this study may be biased because they represent only a major disease among older adults.

## Conclusions

Our study demonstrates the level of total care expenditures and the characteristics that influence the total expenditure on health care and LTC services. The total care expenditures of older adults are approximately 1.72 times higher for the LTC services compared to health care services, and the expenditures on personal payments were similar for the two services. The increase in total expenditures was associated with older adults who were female, between 74–84 years of age, and with a higher care needs level. Moreover, factors like disability and living alone resulted in the older person spending less on care services, while older adults who suffered from diseases and lived in residential LTC facilities spent more money on these services. We should continue to identify and focus on the factors involved in providing services for older individuals and complement the lack of services or inadequate services to enhance the LTC and health care service systems. With the increase in the older adult population, there is a need for corresponding changes in the care system and, at the same time, dealing with financial challenges that require high expenditure. It is essential to ensure that the care system is sustained through a wide variety of research efforts.

## Data Availability

The data that support the findings of this study are available from the National Health Insurance Service but restrictions apply to the availability of these data, which were used under license for the current study, and so are not publicly available. Data are however available from the authors upon reasonable request and with permission from the National Health Insurance Service.

## Abbreviations

ANOVA: Analysis of variance
LSD: Least significant difference
LTC: Long-term care
LTCI: Long-term care insurance
GDP: Gross Domestic Product
NHIS: National Health Insurance Service
OECD: Organisation for Economic Co-operation and Development
USD: United States Dollar
